# Tumor Response Predicts Survival Time of Nivolumab Monotherapy for Advanced Gastric Cancer: A Subgroup Analysis of the DELIVER Trial (JACCRO GC-08)

**DOI:** 10.1093/oncolo/oyae056

**Published:** 2024-04-06

**Authors:** Yu Sunakawa, Yasuhiro Sakamoto, Ryohei Kawabata, Atsushi Ishiguro, Yusuke Akamaru, Yosuke Kito, Masazumi Takahashi, Jin Matsuyama, Hiroshi Yabusaki, Akitaka Makiyama, Takahisa Suzuki, Masahiro Tsuda, Hisateru Yasui, Jun Hihara, Atsushi Takeno, Eisuke Inoue, Wataru Ichikawa, Masashi Fujii

**Affiliations:** Department of Clinical Oncology, St. Marianna University School of Medicine, Kawasaki, Japan; Department of Medical Oncology, Osaki Citizen Hospital, Osaki, Japan; Department of Surgery, Sakai City Medical Center, Sakai, Japan; Department of Medical Oncology, Teine Keijinkai Hospital, Sapporo, Japan; Department of Gastrointestinal Surgery, Ikeda City Hospital, Ikeda, Japan; Department of Medical Oncology, Ishikawa Prefectural Central Hospital, Kanazawa, Japan; Division of Gastroenterological Surgery, Yokohama Municipal Citizen’s Hospital, Yokohama, Japan; Department of Gastroenterological Surgery, Higashi-Osaka City Medical Center, Higashi-Osaka, Japan; Department of Gastroenterological Surgery, Niigata Cancer Center Hospital, Niigata, Japan; Cancer Center, Gifu University Hospital, Gifu, Japan; Department of Surgery, National Hospital Organization Kure Medical Center and Chugoku Cancer Center, Kure, Japan; Department of Gastroenterolgical Oncology, Hyogo Cancer Center, Akashi, Japan; Deparment of Medical Oncology, Kobe City Medical Center General Hospital, Kobe, Japan; Department of Surgery, Hiroshima City North Medical Center Asa Citizens Hospital, Hiroshima, Japan; Department of Surgery, Kansai Rosai Hospital, Amagasaki, Japan; Showa University Research Administration Center, Showa University, Tokyo, Japan; Division of Medical Oncology, Showa University Fujigaoka Hospital, Yokohama, Japan; Japan Clinical Cancer Research Organization (JACCRO), Tokyo, Japan

**Keywords:** gastric cancer, nivolumab, tumor response, real-world data

## Abstract

**Background:**

This prospective observational study evaluated the real-world effectiveness of nivolumab monotherapy in previously treated advanced gastric cancer (GC). A preplanned 2-year final analysis was performed to confirm survival and tumor behavior with nivolumab monotherapy.

**Patients and Methods:**

The primary endpoint was overall survival (OS). The data regarding tumor size were prospectively collected and evaluated using the RECIST criteria. Exploratory analyses were performed for survival according to the tumor response and depth of response (DpR) in patients with measurable lesions who were receiving nivolumab monotherapy as third- or later-line therapy.

**Results:**

In 487 patients, the median OS and progression-free survival (PFS) were 5.8 (95% CI 5.3-6.9) months and 1.8 (95% CI 1.7-2.0) months, respectively. The response rate (RR) was 14.5% in 282 patients with measurable lesions. In 234 patients treated with third- or later-line, the DpR was found to be associated with PFS and OS in the Spearman analysis (*r* = 0.55 and 0.44, respectively) as well as using a discrete variable. When the DpR was divided into 5 groups (−20%≥DpR; −20%<DpR ≤ 0%; 0%<DpR ≤ 30%; 30%<DpR ≤ 50%; 50%<DpR) according to tumor shrinkage, clinically meaningful differences in PFS, and OS were noted. Patients with DpR of ≥30% had favorable survival time in nivolumab monotherapy as a later-line treatment.

**Conclusion:**

The final analysis confirmed the efficacy of nivolumab monotherapy for patients with advanced GC in routine clinical practice. The exploratory analysis indicated that increasing DpR was associated with longer median PFS and OS in nivolumab treatment at a later-line setting.

## Teaser

This study evaluated the real-world effectiveness of nivolumab monotherapy in previously treated advanced gastric cancer. A preplanned 2-year final analysis was performed to confirm survival and tumor behavior with nivolumab monotherapy.

**Implications for Practice**

Nivolumab monotherapy is clinically effective for patients with advanced gastric cancer in routine clinical practice. No associations were found between clinical outcomes and tumor burden/tumor growth rate before nivolumab monotherapy. Increasing depth of response was associated with a longer survival time of nivolumab monotherapy at later-line setting.

## Introduction

Nivolumab has become the standard of care for later-line treatment in advanced gastric cancer (GC) and offers a prognostic benefit in patients with GC without biomarker selection. The ATTRACTION-2 trial reported landmark results showing that nivolumab is effective in previously treated advanced or recurrent GC.^1^ In that trial, patients were randomized into the nivolumab monotherapy group and the placebo group, revealing significantly longer survival in the nivolumab monotherapy group, with overall survival (OS) as the primary endpoint. In clinical practice, real-world data (RWD) of nivolumab in the later-line treatment of advanced GC are important, and our observational study of nivolumab monotherapy, the DELIVER trial, showed response rate (RR), progression-free survival (PFS), and OS similar to the results of the ATTRACTION-2 trial, presenting that nivolumab monotherapy would be as effective in clinical practice as in clinical trials.^2^ However, our study had a relatively short median follow-up period of 20.5 months, and further analysis is needed.

Although nivolumab monotherapy in later-line treatment for advanced GC does not have a high RR, a subgroup analysis of clinical trials revealed a good prognosis in patients who achieved a tumor reduction greater than partial response (PR).^3,4^ Although nivolumab is currently used in combination with chemotherapy for first-line treatment, the analysis of the relationship between tumor shrinkage and prognosis with nivolumab monotherapy would provide clinically helpful data for its use in first-line treatment. However, studies analyzing the correlation between tumor shrinkage and prognosis with nivolumab monotherapy in the later-line setting are limited. In colorectal cancer, tumor shrinkage with chemotherapy has been shown to correlate with prognosis, and this information would be useful in clinical practice.^5-7^

Thus, we performed a preplanned prognostic updated analysis of the DELIVER trial 2 years from the last patient enrollment and further evaluated the correlation between tumor shrinkage with nivolumab and prognosis in patients with advanced GC.

## Methods

### Study Design

The DELIVER trial (Japan Clinical Cancer Research Organization (JACCRO) GC-08; UMIN000030850) was a multicenter, prospective observational, and translational study aimed to evaluate the efficacy and toxicity of nivolumab monotherapy in patients with advanced GC and identify novel predictors for nivolumab. The institutional review board at each institution approved the study protocol, and the study was conducted in accordance with the principles of the Declaration of Helsinki and Ethical Guidelines for Medical and Health Research Involving Human Subjects. After enrollment, patients were prospectively monitored for clinical outcomes by the termination of nivolumab monotherapy, and imaging was performed regularly according to physicians’ decisions. All participating patients provided written informed consent before study enrollment. Data were maintained by the independent JACCRO GC-08 Data Center and were analyzed by the JACCRO Statistical Analysis Department. Data and analyses were verified and assured by all academic members of the steering committee.

### Study Endpoints

The primary endpoint of this study was OS. Secondary endpoints included RR, disease control rate (DCR), PFS, tumor regression rate (TRR), tumor progression rate (TPR), and safety. TRR was the tumor decrease rate from baseline in patients with tumor regression, while TPR was the tumor increase rate from baseline in patients with tumor progression. The frequency of the worst grade in all courses was calculated for each adverse event according to the Common Terminology Criteria for Adverse Events ver. 4.0. In this updated analysis, the relationship between tumor shrinkage with nivolumab and survival time was evaluated.

### Study Population

This study enrolled patients who met the following criteria: (1) patients with advanced or recurrent unresectable GC or gastroesophageal junction cancer that was histologically confirmed to be adenocarcinoma, (2) patients who will receive nivolumab 240 mg/body biweekly, (3) patients with an Eastern Cooperative Oncology Group performance status (PS) of 0-2, (4) patients aged ≥ 20 years at the time of providing informed consent, (5) patients who could submit fecal samples and undergo blood collection before the start of nivolumab monotherapy and when becoming refractory/intolerant to the treatment, and (6) patients who were fully informed regarding the content of this study and personally provided written consent to participate in the study.

The ineligibility criteria were as follows: patients who had previously received nivolumab monotherapy, patients with hepatitis B surface antigen or hepatitis C virus antibody, and patients considered by the investigator or sub-investigator to be inappropriate to participate in this study.

### Statistical Design

Efficacy and safety analysis sets consisted of patients enrolled in this study and who received at least one course of nivolumab. OS and PFS were analyzed based on the efficacy analysis set and estimated using the Kaplan-Meier (K-M) method. The RR was analyzed based on the efficacy analysis set with measurable lesions. The tumor growth rate (TGR) was defined as the percentage increase in the tumor volume between 2 imaging measurements in 1 month according to previous reports.^8,9^ The TGR was calculated by the sum of the diameter of the target lesions according to RECIST criteria and the time interval between the 2 imaging assessments to compare the growth rate before and after nivolumab monotherapy.

The association between TGR or tumor burden and survival time was analyzed using Spearman’s rank correlation coefficient. In addition, the depth of response (DpR), which was defined as the percentage of tumor shrinkage, was explored based on the longitudinal diameters of the target lesions according to the RECIST criteria at the lowest point (nadir) compared with baseline values. Also, DpR was evaluated either as continuous or ordinal variable, with 5 levels based on quintile distribution. The associations between tumor response/DpR and survival time were analyzed. The significance level was set to .05. Analyses were performed with R version 4.0.3 (R Core Team [2020], R Foundation for Statistical Computing, Vienna, Austria. URL https://www.R-project.org/).

The cutoff date for the current analysis was August 2, 2021, at the time of 2 years after the last enrollment, which was preplanned as the final analysis.

## Results

### Patient Characteristics

This study enrolled 501 patients, from 67 institutions, between March 2018 and August 2019, and 487 patients were evaluable with clinical data. The patient cohort with measurable lesions consisted of 282 patients, and 219 patients were included in the cohort evaluable for TGR (Supplementary Fig. S1). The characteristics of the 487 evaluable patients are summarized in Supplementary Table S1. At the time of data analysis, the median follow-up time was 32.4 (range 24.5-41.2) months. In addition, 67 (14%) of 487 patients had a PS of 2, and ascites and peritoneal metastases were observed in 206 (42%) and 227 (47%) patients, respectively.

In total, 474 patients discontinued the protocol treatment, and 13 patients continued to receive treatment at the data cutoff of August 2021. The median number of cycles administered per patient was 4 (range, 1-73). During analysis, the mean relative dose intensity of nivolumab was 89.2%. Post-treatment with chemotherapy after nivolumab monotherapy was administered to 213 of 487 patients and 213 patients, in which post-treatment regimens included irinotecan (*n* = 94, 44.1%), trifluridine/tipiracil (*n* = 29, 13.6%), irinotecan plus ramucirumab (*n* = 14, 6.6%), and nivolumab (*n* = 13, 6.1%).

### Efficacy and Safety

In the survival analysis based on 433 OS events and 468 PFS events, the median OS was 5.82 (95% CI 5.29-6.87) months, and the median PFS was 1.84 (95% CI, 1.71-1.97) months. In 282 patients with measurable lesions, the RR was 14.5% (95% CI 10.6%-19.2%) with a complete response (CR) of 2.1% and PR of 12.4%. The DCR was 39.4% (95% CI 35.1-43.9) in 487 evaluable patients. Safety was assessed in 487 patients in the safety population who received at least one course of nivolumab. The overall incidences of hematological and non-hematological toxicities are shown in Supplementary Table S2. No unexpected toxicities were observed in the safety population.

### Association Between the TGR or Tumor Burden and Clinical Outcomes

**Figure 1.**
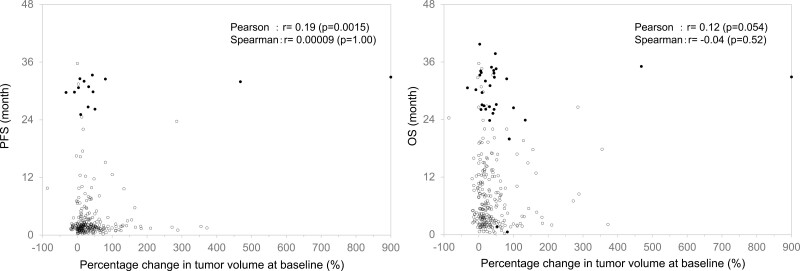
Association between survival time and change in the tumor volume at baseline. Left, progression-free survival; right, overall survival.

**Figure 2.**
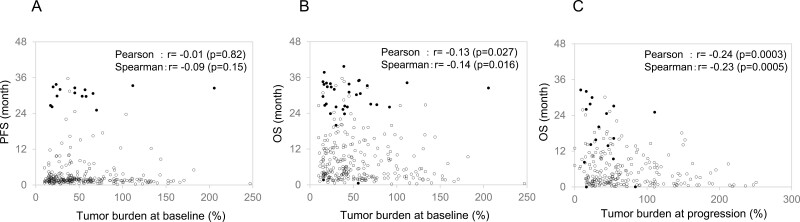
Association between survival time and tumor burden at each time point. (**A**) Progression-free survival at baseline, (**B**) overall survival at baseline, and (**C**) overall survival at disease progression.

The median TRR and TPR were 23.9% and 38.9%, in 75 patients with tumor regression and 185 with tumor progression, respectively. The TGR decreased from 3.8 to 2.8 mm/month after nivolumab initiation. The TGR at pretreatment did not correlate with PFS and OS (Fig. 1). No associations were found between the PFS/OS and tumor burden at baseline before nivolumab monotherapy. In addition, no association was observed between the survival time and tumor burden at disease progression after nivolumab monotherapy (Fig. 2).

### Clinical Outcomes According to Tumor Response or DpR

**Figure 3.**
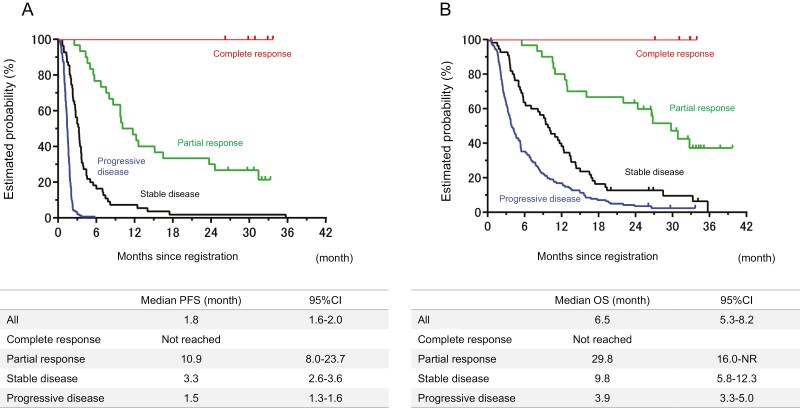
Survival time by tumor response. (**A**) Progression-free survival and (**B**) overall survival.

The exploratory analysis was performed to evaluate the effect of tumor shrinkage of nivolumab monotherapy on survival time in 234 patients with measurable lesions who received nivolumab as a third- or later-line treatment (Supplementary Fig. S1). The median PFS and OS were not reached in patients with CR, 11.7 and 29.8 months in patients with PR, 3.8 and 11.4 months in patients with stable disease, and 1.5 and 4.2 months in patients with progressive disease (PD; Fig. 3).

**Figure 4.**
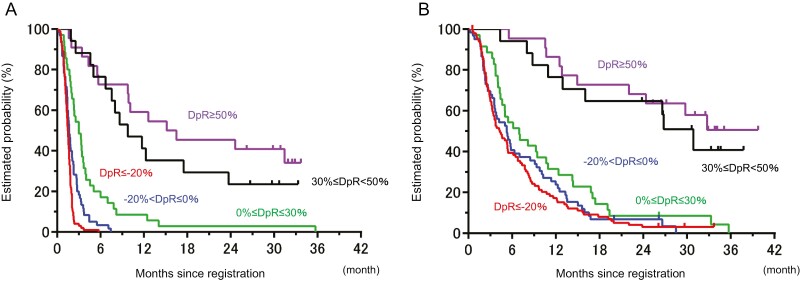
Survival time by depth of response. (**A**) Progression-free survival and (**B**) overall survival.

In addition, the median DpR was −14.1% and was associated with PFS and OS in the Spearman analysis (*r* = 0.55 and 0.44, respectively; Supplementary Fig. S2). The analysis of distribution of DpR indicated that a highly significant association of DpR, as a discrete variable, with PFS and OS was found (Supplementary Fig. S3). Moreover, we divided into 5 groups according to tumor shrinkage to generate clinically useful data for nivolumab treatment (−20%≥DpR; −20%<DpR ≤ 0%; 0%<DpR ≤ 30%; 30%<DpR ≤ 50%; 50%<DpR). This exploratory approach found a clinically meaningful difference in PFS and OS. Patients with DpR of ≥30% had favorable survival time in nivolumab monotherapy as a later-line treatment (Fig. 4; Table 1).

**Table 1.** Survival time according to depth of response.

	Median PFS (months)	HR, *P*-value	Median OS (months)	HR, *P*-value
DpR≤−20% (*n* = 100)	1.5	1	4.3	1
−20%<DpR ≤ 0% (*n* = 60)	1.7	0.57, .0011	5.3	0.89, .50
0%<DpR < 30% (*n* = 35)	3.0	0.24, <.0001	7.0	0.68, .052
30%≤DpR < 50% (*n* = 17)	9.8	0.043, <.0001	30.9	0.17, <.0001
DpR ≥ 50% (*n* = 22)	15.8	0.049, <.0001	Not reached	0.14, <.0001

Abbreviations: OS, overall survival; PFS, progression-free survival.

## Discussion

The DELIVER trial was designed to establish RWD for and validate biomarkers of nivolumab monotherapy.^10^ In this preplanned updated analysis, the follow-up period was extended, and the analysis was performed after a sufficient number of OS events had occurred; however, the results showed a treatment response similar to that in the clinical trial, confirming that nivolumab monotherapy is effective enough to be used in routine clinical practice. The results also provided valuable data on the correlation between tumor shrinkage and prognosis with nivolumab monotherapy.

This study demonstrated that nivolumab monotherapy is effective regardless of the size of the tumor before treatment or the speed of tumor growth. Several studies revealed that fast-growing tumors or large tumors are less likely to respond to immune checkpoint inhibitors.^11,12^ In GC, the efficacy of nivolumab monotherapy in such cases is a concern. Therefore, we analyzed the speed of tumor growth and tumor volume immediately before nivolumab therapy and its correlation with prognosis after nivolumab monotherapy. Our results indicate that pretreatment tumor status may not be a predictor of response to nivolumab monotherapy and that in cases where immune checkpoint inhibitors were not used in first-line therapy, they must be used robustly post-treatment as a standard of care.

In the analysis of nivolumab combination therapy in the first-line treatment of GC, patients with good tumor reduction or shrinkage have a good prognosis according to the CheckMate 649 trial.^13^ However, few data reveal the association between tumor response and survival time in later-line treatment with nivolumab alone for advanced GC. Therefore, we performed post hoc exploratory analyses of survival time by tumor response from the DELIVER trial. The CR or PR group had a better prognosis, with a significant difference in survival compared with the SD or PD groups, with a trend toward a better prognosis than PD if SD was obtained. In the DpR analysis, approximately 32% of the patients showed tumor shrinkage from the baseline. The analysis by distribution of DpR showed a significant association between DpR and PFS/OS in this study. However, the median DpR for V quintile was 22%, and many cases other than V quintile had a low DpR and included cases with tumor enlargement. The K-M curves for I-IV quintile were not far apart and no differences were observed. Therefore, we evaluated survival time in 5 categories according to DpR in order to construct clinically useful data. When the DpR was divided into 5 groups according to tumor shrinkage, a significant difference in PFS and OS was found; thus, increasing DpR was associated with longer median PFS and OS. The results of these analyses may be useful in predicting the prognosis of patients who experience tumor shrinkage with nivolumab in routine clinical practice. If the prognosis after treatment could be predicted, it would be helpful to consider when to switch to a post-treatment. Nivolumab was also found to correlate with tumor shrinkage and prognosis in GC post-treatment. A study reported that DpR correlates with survival differently for different antibody drugs.^14^ Thus, analyzing data from more clinical trials in the future may be worthwhile to see if DpR is a surrogate for survival in immunotherapy.

This study had some limitations. We could show a correlation between tumor shrinkage and prognosis with nivolumab in patients with advanced GC with later-line treatment. However, the imaging studies performed to assess tumor shrinkage were not planned in the protocol but were routinely performed by the investigators in routine clinical practice. This analysis was completely exploratory and did not show a prospective correlation between tumor shrinkage and prognosis. Therefore, a prospective study is warranted to confirm our results. In addition, tumor shrinkage is a post-treatment event and cannot be predicted pretreatment; thus, it is not a specific biomarker for drug selection. Moreover, biomarkers that can predict which patients will achieve tumor shrinkage from nivolumab must be developed. Biomarker research accompanies this study, and biomarker development is being conducted using blood and stool samples. Further analysis is needed to discover biomarkers that can predict tumor shrinkage with nivolumab.

## Conclusion

The updated analysis confirmed the efficacy of nivolumab for patients with advanced GC in routine clinical practice, with comparable outcomes to previous clinical trials. Nivolumab was effective regardless of tumor burden or TGR pretreatment in advanced GC. In addition, the exploratory analysis indicated the association between DpR and survival time in patients with advanced GC treated with nivolumab monotherapy at a later-line setting. Moreover, an increasing DpR was associated with longer median PFS and OS.

## Supplementary Material

oyae056_suppl_Supplementary_Figures

oyae056_suppl_Supplementary_Tables_S1

oyae056_suppl_Supplementary_Tables_S2

## Data Availability

The dataset analyzed during the current study are available from the corresponding author on reasonable request (jaccro@jaccro.or.jp).
