# Supplementary material for: Tumor Response Predicts Survival Time of Nivolumab Monotherapy for Advanced Gastric Cancer: A Subgroup Analysis of the DELIVER Trial (JACCRO GC-08)
Source: Oncologist. 2024 Apr 6;29(8):e997–e1002. doi: 10.1093/oncolo/oyae056 (PMC11299930; doi:10.1093/oncolo/oyae056)
Supplement: oyae056_suppl_Supplementary_Figures [file oyae056_suppl_supplementary_figures.zip › Supplementary Figure Captions.docx]

**Supplementary Figure Captions**

**Supplementary Figure 1.** Patient flow

**Supplementary Figure 2.** Water-fall plot

**Supplementary Figure 3.** Association of DpR and survival time by exploratory analysis using a discrete variable
