# Supplementary figures and images for: Tumor Response Predicts Survival Time of Nivolumab Monotherapy for Advanced Gastric Cancer: A Subgroup Analysis of the DELIVER Trial (JACCRO GC-08)
Source: Oncologist. 2024 Apr 6;29(8):e997–e1002. doi: 10.1093/oncolo/oyae056 (PMC11299930; doi:10.1093/oncolo/oyae056)

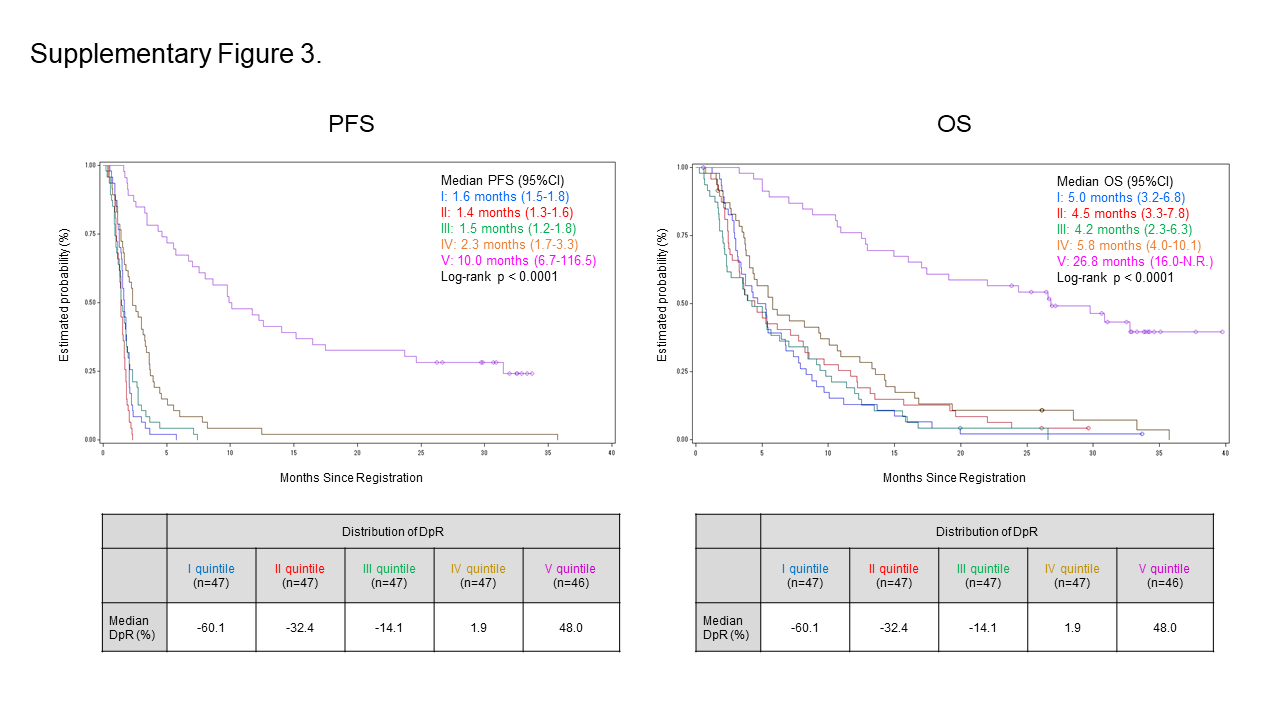

Supplement: oyae056_suppl_Supplementary_Figures [file oyae056_suppl_supplementary_figures.zip › Supplementary Figure 3.tif]

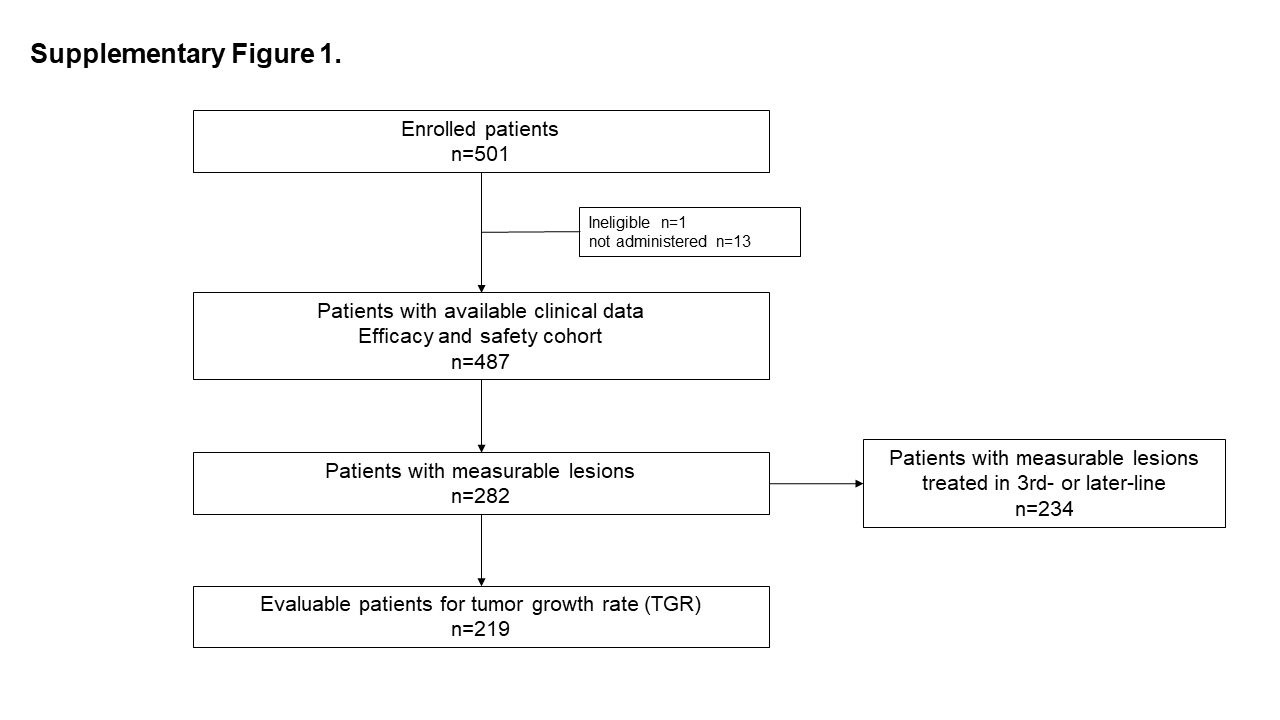

Supplement: oyae056_suppl_Supplementary_Figures [file oyae056_suppl_supplementary_figures.zip › Supplementary Figure 1.tif]

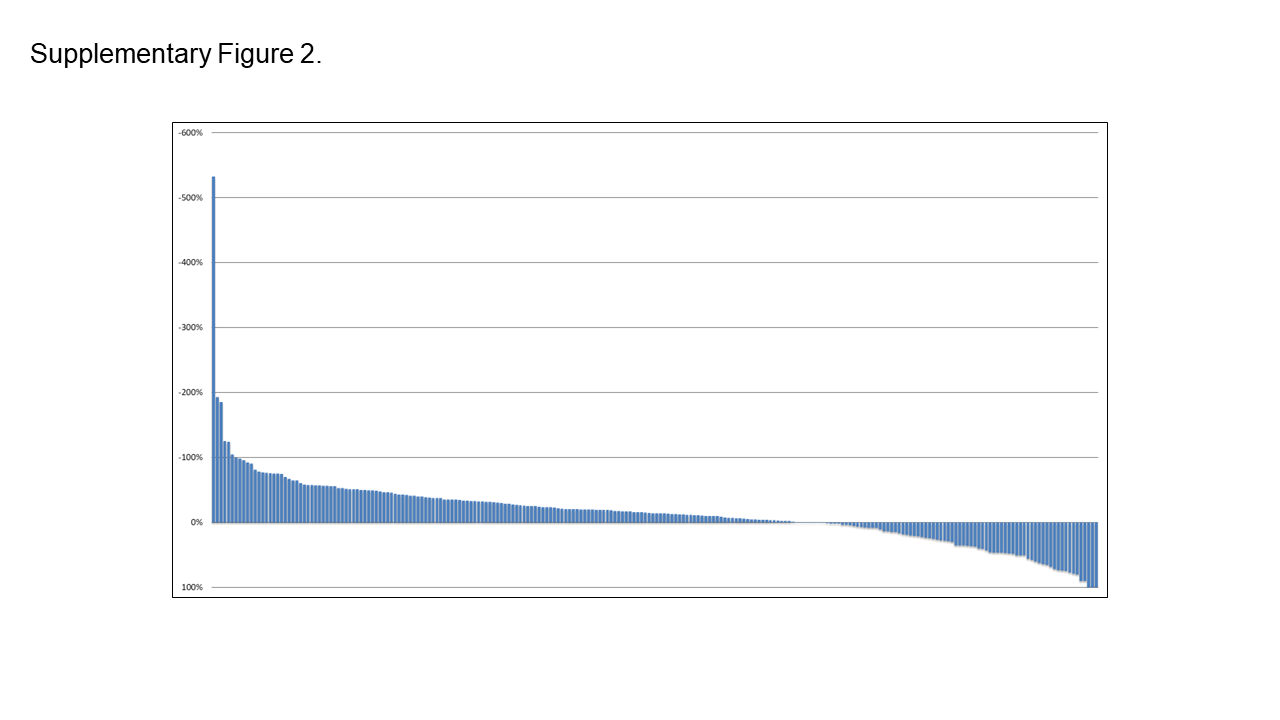

Supplement: oyae056_suppl_Supplementary_Figures [file oyae056_suppl_supplementary_figures.zip › Supplementary Figure 2.tif]
