# Supplementary material for: Tumor Response Predicts Survival Time of Nivolumab Monotherapy for Advanced Gastric Cancer: A Subgroup Analysis of the DELIVER Trial (JACCRO GC-08)
Source: Oncologist. 2024 Apr 6;29(8):e997–e1002. doi: 10.1093/oncolo/oyae056 (PMC11299930; doi:10.1093/oncolo/oyae056)
Supplement: oyae056_suppl_Supplementary_Tables_S1 [file oyae056_suppl_supplementary_tables_s1.docx]

**Supplementary Table 1. Patient characteristics.**

| **Patient characteristics** | **Total (n = 487)** |
| --- | --- |
| Sex, male/female, n (%) | 347 (71.3%)/140 (28.7%) |
| Age (years)  Median (range) | 70 (26–90) |
| ECOG PS 0/1/2, n (%) | 206 (42.3%)/214 (43.9%)/67 (13.8%) |
| Body mass index  Median (range) | 20.6 (12.9–36.2) |
| Diagnosis  Advanced/relapse, n (%) | 308 (66.2%)/179 (33.8%) |
| Previous gastrectomy  Yes/no, n (%) | 243 (49.9%)/244 (50.1%) |
| Number of previous regimens  ≤1/2/≥3, n (%) | 74 (15.2%)/274(56.3%)/139 (28.5%) |
| Ascites  Yes/no, n (%) | 206 (42.3%)/281 (57.7%) |
| Peritoneal metastasis  Yes/no, n (%) | 227 (46.6%)/260 (53.4%) |
| Histological type, n (%)  Non-diffuse  Diffuse  Unknown | 221 (45.4%)  227 (46.6%)  39 (8.0%) |
| HER2 status  Positive/negative/unknown, n (%) | 101 (20.7%)/348 (71.5%)/38 (7.8%) |
| Previous therapies, n (%)  Taxane  Ramucirumab  Irinotecan | 447 (91.8%)  403 (82.8%)  49 (10.1%) |

ECOG, Eastern Cooperative Oncology Group; PS, performance status; n, number.
