# Supplementary material for: Tumor Response Predicts Survival Time of Nivolumab Monotherapy for Advanced Gastric Cancer: A Subgroup Analysis of the DELIVER Trial (JACCRO GC-08)
Source: Oncologist. 2024 Apr 6;29(8):e997–e1002. doi: 10.1093/oncolo/oyae056 (PMC11299930; doi:10.1093/oncolo/oyae056)
Supplement: oyae056_suppl_Supplementary_Tables_S2 [file oyae056_suppl_supplementary_tables_s2.docx]

**Supplementary Table 2. Adverse events occurring in the safety population (n = 487)**

| **Adverse events** | **Grade 1**  **n (%)** | **Grade 2**  **n (%)** | **Grade 3**  **n (%)** | **Grade 4**  **n (%)** | **≥Grade 1**  **n (%)** | **≥Grade 3**  **n (%)** |
| --- | --- | --- | --- | --- | --- | --- |
| Leukopenia | 18 (3.7) | 18 (3.7) | 4 (0.8) | 1 (0.2) | 41 (8.4) | 5 (1.0) |
| Neutropenia | 37 (7.6) | 22 (4.5) | 9 (1.8) | 0 (0.0) | 68 (14) | 9 (1.8) |
| Anemia | 24 (4.9) | 82 (16.8) | 46 (9.4) | 17 (3.5) | 169 (34.7) | 63 (12.9) |
| Platelet count decreased | 62 (12.7) | 10 (2.1) | 14 (2.9) | 3 (0.6) | 89 (18.3) | 17 (3.5) |
| Nausea | 64 (13.1) | 39 (8.0) | 15 (3.1) | 0 (0.0) | 118 (24.2) | 15 (3.1) |
| Vomiting | 55 (11.3) | 18 (3.7) | 6 (1.2) | 0 (0.0) | 79 (16.2) | 6 (1.2) |
| Mucositis oral | 16 (3.3) | 5 (1.0) | 1 (0.2) | 0 (0.0) | 22 (4.5) | 1 (0.2) |
| Diarrhea | 63 (13.1) | 18 (3.7) | 6 (1.2) | 0 (0.0) | 87 (17.9) | 6 (1.2) |
| Anorexia | 63 (13.1) | 79 (16.2) | 60 (12.3) | 1 (0.2) | 203 (41.7) | 61 (12.5) |
| Fatigue | 49 (10.1) | 50 (10.3) | 34 (7.0) | 0 (0.0) | 133 (27.3) | 34 (7.0) |
| Malaise | 86 (17.7) | 113 (23.2) |  |  | 199 (40.9) |  |
| AST increased | 115 (23.6) | 46 (9.4) | 38 (7.8) | 5 (1.0) | 204 (41.9) | 43 (8.8) |
| ALT increased | 116 (23.8) | 25 (5.1) | 15 (3.1) | 2 (0.4) | 158 (32.4) | 17 (3.5) |
| Hyponatremia | 158 (32.4) | 0 (0.0) | 27 (5.5) | 2 (0.4) | 187 (38.4) | 29 (6.0) |
| Hypokalemia | 67 (13.8) | 0 (0.0) | 16 (3.3) | 2 (0.4) | 85 (17.5) | 18 (3.7) |
| Pyrexia | 47 (9.7) | 16 (3.3) | 2 (0.4) | 0 | 65 (13.3) | 2 (0.4) |
|  |  |  |  |  |  |  |
| Hypothyroidism | 13 (2.7) | 16 (3.3) | 1 (0.2) | 0 (0.0) | 30 (6.2) | 1 (0.2) |
| Hyperthyroidism | 2 (0.4) | 6 (1.2) | 0 (0.0) | 0 (0.0) | 8 (1.6) | 0 (0.0) |
| Interstitial lung disease | 3 (0.6) | 8 (1.6) | 6 (1.2) | 0 (0.0) | 17 (3.5) | 6 (1.2) |
| Colitis | 0 (0.0) | 6 (1.2) | 2 (0.4) | 0 (0.0) | 8 (1.6) | 2 (0.4) |
| Maculopapular rash | 4 (0.8) | 1 (0.2) | 0 (0.0) | 0 (0.0) | 5 (1.0) | 0 (0.0) |
| Hypophysitis | 1 (0.2) | 1 (0.2) | 0 (0.0) | 0 (0.0) | 2 (0.4) | 0 (0.0) |

ALT, alanine transaminase; AST, aspartate transaminase
